# Identification and validation of mitochondrial transport and glycolysis-associated prognostic and therapeutic target for lung adenocarcinoma patients

**DOI:** 10.3389/fgene.2026.1924235

**Published:** 2026-08-27

**Authors:** Yihang Gu, Minhua Shi

**Affiliations:** 1 Department of Respiratory and Critical Care Medicine, Second Affiliated Hospital of Soochow University, Suzhou, Jiangsu, China; 2 Department of Gerontology, Jiangyin People’s Hospital, Jiangyin, Jiangsu, China

**Keywords:** experimental validation, glycolysis, lung adenocarcinoma, machine learning, mitochondrial transport, multi-omics

## Abstract

**Background:**

Lung adenocarcinoma (LUAD) remains a leading cause of cancer mortality, with metabolic reprogramming, particularly dysregulated mitochondrial transport and glycolysis (MG), emerging as a hallmark of its progression. Despite the recognized importance of these pathways, an integrated, clinically actionable model that combines them for patient stratification and therapeutic targeting is lacking.

**Objective:**

This study aims to decode integrated MG-associated molecular patterns in the progression of LUAD, providing novel clinical actionable target for LUAD patients.

**Methods:**

GSVA and WGCNA were applied to an integrated GEO cohort to identify MG-associated genes. LASSO-Cox and multivariable Cox analyses were used to construct and validate a prognostic model and prioritize CCNA2 as a prognostic candidate. Single-cell, spatial, cell-based, drug-sensitivity, and xenograft analyses were used for validation.

**Results:**

The MG-associated signature stratified overall survival, and CCNA2 was enriched in malignant cells. CCNA2 knockdown reduced A549 proliferation, wound closure, and invasion. GSCA analysis identified BHG712, IPA-3, and KIN001-260 as candidate compounds, and IPA-3 produced the largest reduction in xenograft tumor burden.

**Conclusion:**

The integrated analyses identify an MG-associated prognostic framework and prioritize CCNA2 for further validation in LUAD.

## 1 Introduction

Lung adenocarcinoma (LUAD), the predominant histological subtype of non-small cell lung cancer (NSCLC), accounts for approximately 40% of all lung cancer diagnoses and represents a leading cause of cancer-related mortality worldwide (Wei et al., 2023). Despite significant advancements in targeted therapies and immunotherapies in the past decade, the overall 5-year survival rate for LUAD patients remains dismally below 20%, largely due to late-stage diagnosis and the inevitable emergence of therapeutic resistance (Shen et al., 2026; Deng et al., 2021). Genomic alterations in key oncogenes, such as EGFR, KRAS, and ALK have been well-characterized (Sun et al., 2024). However, the profound metabolic reprogramming that sustains tumor growth and invasion, particularly the interplay between mitochondrial transport and glycolysis(MG), remains a fertile area for identifying novel prognostic biomarkers and therapeutic vulnerabilities (Huang et al., 2024).

Dysregulated metabolism is a hallmark of cancer (Chang et al., 2022). In LUAD, a metabolic shift known as aerobic glycolysis, or the Warburg effect, is a central pathogenic feature, of which cancer cells preferentially convert glucose to lactate even in the presence of oxygen (Shen et al., 2026; Jin et al., 2024). This is driven by aberrant activation of key glycolytic enzymes like HK2 and PKM2, fueling anabolic biosynthesis and maintaining redox balance (Zhou Y. et al., 2024). Concurrently, mitochondrial transport, encompassing the shuttling of metabolites, ions, and proteins across the mitochondrial membranes, is intricately rewired in LUAD (Dias Amoedo et al., 2020). For instance, the calcium uniporter MCU complex modulates mitochondrial calcium uptake, which is essential for regulating oxidative phosphorylation (OXPHOS) and cell death, and its dysregulation is linked to lung cancer cell proliferation and metastasis (Wei et al., 2025). Furthermore, the interplay between glycolysis and mitochondrial function is not merely a simple switch, it is rather than a complex, dynamic crosstalk exists. The glycolytic intermediate pyruvate can fuel the tricarboxylic acid (TCA) cycle, while mitochondrial metabolites can feedback to regulate glycolysis (Passarella et al., 2021). This metabolic codependency suggests that a combined signature of MG may more accurately capture the metabolic state driving LUAD progression than either pathway alone (Fernie et al., 2004). However, a comprehensive, multi-omic integration of this MG-associated signature for prognostic stratification and therapeutic target discovery in LUAD is currently lacking.

To bridge this knowledge gap, we conducted a systematic, machine learning-driven multi-omic study. We first identified a robust MG-associated gene signature in LUAD patients using GSVA and WGCNA. Subsequently, we constructed and validated a prognostic model based on this signature across multiple large-scale cohorts. Through integrative analysis, we pinpointed CCNA2 as a Cox-prioritized prognostic candidate. We further characterized its spatial and temporal dynamics at the single-cell level, elucidated its role in malignant cell progression, and identified potential therapeutic agents, culminating in experimental validation. This study aims to provide a molecular framework that bridges metabolic dysregulation with clinical outcomes, offering new avenues for personalized risk assessment and targeted intervention in LUAD.

## 2 Materials and methods

### 2.1 Bulk data source and processing

Bulk transcriptomic data were obtained from GSE17475, GSE12667, GSE140797, GSE68465, GSE31210, and TCGA-LUAD (Wang et al., 2025; Neumann et al., 2010; Ding et al., 2008; Liang et al., 2020; Leek et al., 2012; Wang et al., 2024; Zhang et al., 2023; Shu et al., 2023). GSE17475, GSE12667, and GSE140797 formed the internal cohort after integration with the sva package; TCGA-LUAD was used for model development and expression analyses, and GSE68465 and GSE31210 for external validation. Analytical sample sizes are reported in the relevant figure legends. Expression data were normalized using limma (Ritchie et al., 2015). The MG-related gene list was obtained from GeneCards (relevance score >1), and HPA images were used for protein-expression assessment (Li et al., 2025).

### 2.2 GSVA and WGCNA analysis

GSVA was performed using the GSVA package in R on the internal cohort to calculate an enrichment score for the MG-related gene set in each sample (Hänzelmann et al., 2013). To identify co-expression networks tightly correlated with this MG score, WGCNA was applied using the WGCNA package in R (Langfelder and Horvath, 2008). A soft-thresholding power (β) was selected to achieve a scale-free topology fit index (R^2^) > 0.90, and the power β = 7 (R^2^ = 0.92) was chosen (Langfelder and Horvath, 2008). A gene co-expression network was constructed, and modules were identified via hierarchical clustering with a minimum module size of 60 (Langfelder and Horvath, 2008). The module whose eigengene showed the strongest Pearson correlation with both the MG score and disease status was selected for downstream analysis (Langfelder and Horvath, 2008). Genes from this module were intersected with the curated MG-related gene list to define the MG-associated shared DEGs. Functional similarity was assessed using GOSemSim (Yu et al., 2010). Mutation data were analyzed using maftools (Awasthi et al., 2024), and GO/KEGG enrichment was performed using clusterProfiler with MSigDB gene sets (Yu et al., 2012).

### 2.3 LASSO-Cox regression analysis and selection of a prognostic candidate

Based on the expression of the 14 MG-related shared DEGs, a LASSO-Cox model was constructed in TCGA-LUAD using glmnet (Zhang D. et al., 2025; Liu et al., 2025). The optimal penalty parameter was selected by 10-fold cross-validation using the 1-SE rule (Zhang D. et al., 2025; Zhou et al., 2026). The model was validated in GSE68465 and GSE31210, and patients were stratified by the median risk score (Zhou et al., 2026). Prognostic performance was assessed using Kaplan-Meier and time-dependent ROC analyses; a nomogram and calibration curves were generated in TCGA-LUAD. Multivariable Cox regression prioritized CCNA2 as a prognostic candidate among the 14 genes, and co-expression analysis was performed in TCGA-LUAD (Meng et al., 2024).

### 2.4 Single-cell and spatial transcriptomic analysis

The LUAD scRNA-seq analysis used three specimens from GSE131907 (Hirano et al., 2026). Specimens were treated as biological units and individual cells as nested analytical observations; panel-specific cell counts are reported in Figures 3, 4. Cells with fewer than 200 or more than 5,000 detected features or mitochondrial-gene percentages above 20% were excluded. Data were normalized using SCTransform, followed by principal-component analysis, clustering at resolution 0.5, and t-SNE/UMAP visualization. Cell types were annotated using SingleR with the Human Primary Cell Atlas reference (Cao et al., 2023). CellChat was used to infer ligand-receptor communication, AUCell to score mitochondrial-transport activity, and scMetabolism to assess glycolytic activity (Kang et al., 2024). Virtual CCNA2 knockout was simulated in the malignant-cell subset using scTenifoldKnk, and Monocle2 was used for trajectory analysis (Osorio et al., 2022). Spatial transcriptomic data from two specimens were processed using Seurat and SCTransform.

### 2.5 Drug enrichment analysis

To identify potential therapeutic agents associated with CCNA2, we used the GSCA drug-sensitivity module to examine Spearman correlations between CCNA2 mRNA expression and drug response (Liu et al., 2023; Su et al., 2024). The analytical N is reported in Figure 6.

### 2.6 Cell lines and culture conditions

The A549 (human LUAD), BEAS-2B (normal lung epithelium), and HEK293T cell lines were acquired from the ATCC (Manassas, VA, United States). These cells were maintained in RPMI-1640 medium (Gibco, United States) containing 10% fetal bovine serum (FBS, Gibco) and 1% penicillin-streptomycin (Gibco, United States). Cultures were kept at 37 °C in a humidified atmosphere with 5% CO_2_. Medium renewal was performed every 48 h, and subculturing was carried out using 0.25% trypsin-EDTA (Gibco, United States) once confluence reached 80%–90%. For all subsequent assays, only cells in the exponential growth phase were employed.

### 2.7 shRNA for silencing

For silencing CCNA2, three distinct shRNA sequences targeting the human gene, together with a scrambled control, were custom-designed and produced by SUNCELL (China). These oligonucleotides were subsequently inserted into a lentiviral expression plasmid. The resulting constructs were packaged into viral particles in HEK293T cells, and the harvested lentivirus was used to transduce A549 cells, thereby achieving stable knockdown of CCNA2.

The specific shRNA sequences used are as follows (5′-3′):

Sh-Control: GCGGCAAGCGAAACTC GCA
Sh-CCNA2-1:GCTGACCCATACCTCAAGTAT
Sh-CCNA2-2: CCT​TAG​GGA​AAT​GGA​GGT​TAA
Sh-CCNA2-3:CCTCTTGATTATCCAATGGAT

### 2.8 q-RT-PCR quantification

RNA isolation was carried out with TRIzol reagent (Invitrogen, United States) following the supplier’s instructions. The yield and purity of the RNA were determined using a NanoDrop One spectrophotometer (Thermo Fisher Scientific, United States). For reverse transcription, 1 µg of total RNA served as a template, and the complementary DNA was generated with the PrimeScript RT Master Mix (Takara Bio, Japan) in a 20-µL reaction mixture. Quantitative real-time PCR was then conducted using TB Green Premix Ex Taq II (YASEN, China) on a QuantStudio 5 instrument (Applied Biosystems, United States), with each sample analyzed in triplicate. The relative expression levels of target mRNAs were calculated via the 2^^−ΔΔCt^ method, using GAPDH as the endogenous normalization control. The oligonucleotide primers employed are listed below (5′→3′):

CCNA2:
F: TGG​ATG​GCA​GTT​TTG​AAT​CA
R: TAA​GAG​GGC​AAG​CAC​AGG​AG
GAPDH:
F: GGA​GCG​AGA​TCC​CTC​CAA​AAT
R: GGC​TGT​TGT​CAT​ACT​TCT​CAT​GG

### 2.9 CCK-8, wound healing and Transwell assays

For the CCK-8 assay, A549 cells were plated at 3,000 cells per well. Absorbance at 450 nm was measured at 24, 48, 72, and 96 h after adding 10 μL of CCK-8 reagent and incubating for 2 h at 37 °C. For the wound-healing assay, confluent monolayers were scratched with a sterile 200-μL tip, washed with PBS, and maintained in serum-free medium. Images were acquired at 0 and 24 h, and wound closure was calculated as [(initial wound area − residual wound area)/initial wound area] × 100%. For the Transwell invasion assay, 1 × 10^4^ cells were seeded in Matrigel-coated inserts; invaded cells were fixed and stained after 24 h, and the signal was normalized to the control group. Each assay was performed in three independent biological experiments per group; technical replicate measurements were averaged within each experiment.

### 2.10 Western blotting(WB)

Total proteins were isolated from BEAS-2B and A549 cells using RIPA buffer containing protease and phosphatase inhibitors. Protein concentrations were quantified using a BCA assay. Aliquots of 30 μg per lane were resolved by 10% SDS-PAGE and transferred to PVDF membranes. Membranes were blocked in 5% non-fat milk/TBST for 1 h, incubated overnight at 4 °C with antibodies against CCNA2 (1:1,000; Abcam, ab181591) and GAPDH (1:10,000; Abcam, ab181602), and then incubated with HRP-conjugated secondary antibodies for 1 h. Bands were visualized using ECL. Western blotting was repeated in three independent biological experiments; Figures 5E,F shows representative images. Lane-labeled images are provided in Supplementary Figures S3, S4. No inferential test was applied to the representative blots.

### 2.11 Nude mouse xenograft model

Twelve 4-week-old female BALB/c nude mice (16–20 g) were maintained under specific pathogen-free conditions. Each mouse received 5.0 × 10^6^ A549 cells in 100 μL PBS by subcutaneous flank injection. When tumors reached 80–120 mm^3^, mice were randomly allocated to vehicle control, BHG712, IPA-3, or KIN001-260 groups (n = 3 independent mice per group). Compounds were administered intraperitoneally at 10 mg/kg three times weekly for 21 days; controls received 5% DMSO, 40% PEG300, 5% Tween-80, and 50% saline. Tumor dimensions and body weight were recorded twice weekly, and volume was calculated as length × width^2^/2. Caliper measurements were not blinded, and no animals were excluded. Humane-removal criteria were tumor diameter >20 mm, body-weight loss ≥20%, hunched posture, or inability to reach food or water. At the endpoint, mice were deeply anesthetized with ketamine/xylazine and euthanized by cervical dislocation; tumors were excised, weighed, and photographed. Each endpoint measurement represented one mouse.

### 2.12 Statistical analysis

Bioinformatic analyses used R 4.2.2, and wet-laboratory analyses used GraphPad Prism 8.0.2. The cell-based experiments tested whether CCNA2 was elevated in A549 cells and whether its knockdown reduced proliferation, wound closure, and invasion; the xenograft experiment tested whether the selected compounds reduced tumor burden. Biological replicates were independently performed experiments, and technical replicates were averaged before analysis. The analytical unit and N are stated in each relevant figure legend. Two-sided Welch t-tests were used for qPCR, wound-closure, and invasion comparisons. CCK-8 data were evaluated by two-way ANOVA followed by Holm-adjusted Welch comparisons at each time point. Xenograft endpoints were evaluated by one-way ANOVA followed by Holm-adjusted comparisons with control. Ninety-five percent confidence intervals were calculated for mean differences. Data are mean ± SD; two-sided P < 0.05 was considered significant.

## 3 Results

### 3.1 Identification of MG-associated DEGs for LUAD patients

**FIGURE 1:**
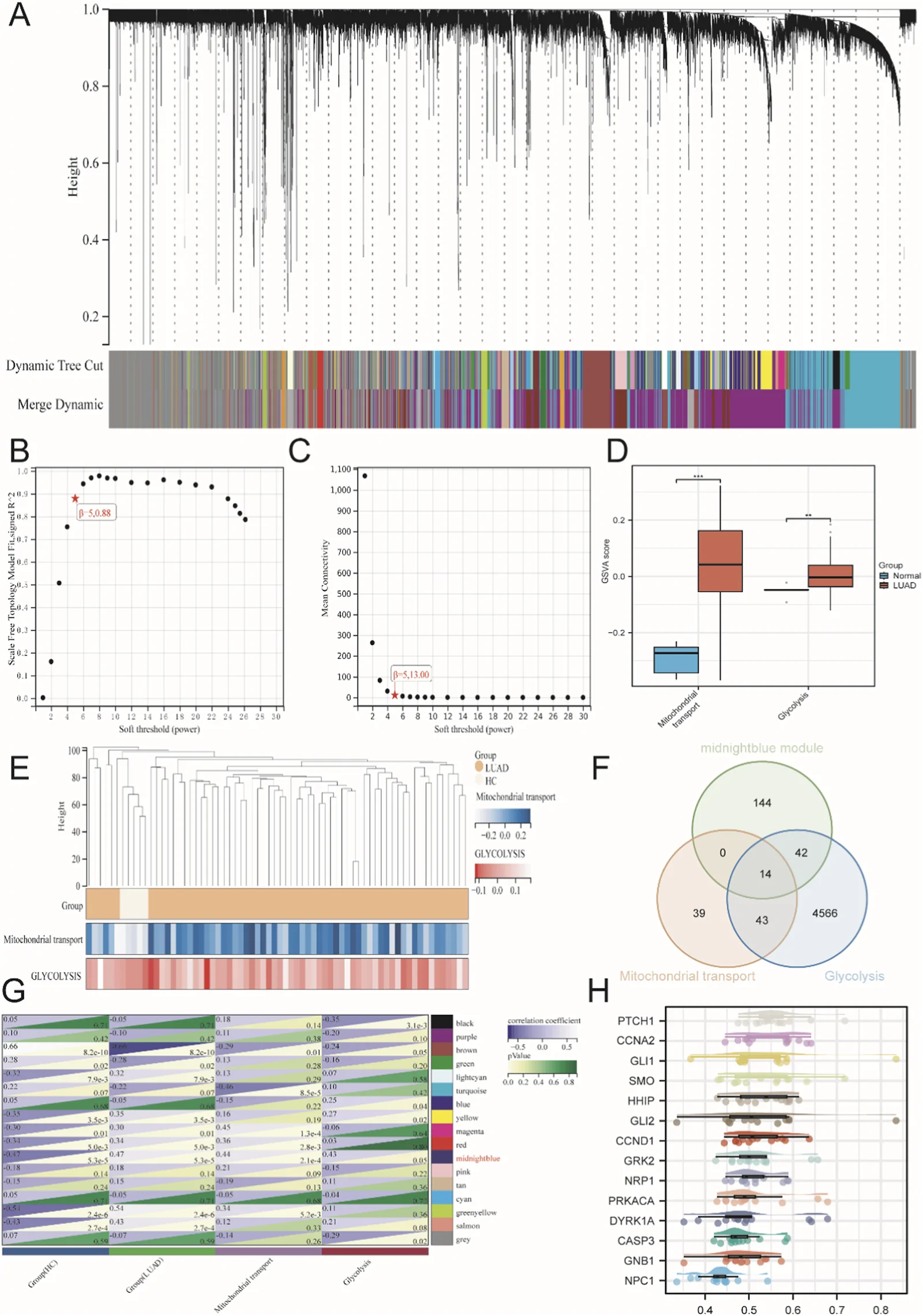
Identification of MG-associated DEGs in LUAD. **(A,E)** WGCNA cluster dendrograms and module assignments. **(B,C)** Soft-threshold selection (β = 5). **(D)** GSVA comparison (n = 7 healthy-control and n = 110 LUAD samples). **(F)** Intersection yielding 14 genes. **(G)** Module-trait correlations in the same 117 samples. **(H)** Functional-similarity analysis. Samples were the analytical units for **(D,G)**.

GSVA score for the MG-related gene set was significantly higher in LUAD samples compared to HC in the combined cohort, indicating the activation of these pathways in LUAD (Figure 1D). WGCNA was then applied to identify co-expression networks correlated with this MG score. A scale-free topology was achieved at β = 5 (Figures 1A–C). Through hierarchical clustering, 17 distinct co-expression modules were identified (Figures 1E,G). The midnightblue module showed the strongest positive correlation with both the MG score and LUAD disease status (Figure 1G). Intersection of the genes in this module with the curated MG-associated gene list yielded 14 MG-associated shared DEGs (Figure 1F). Friend analysis indicated strong functional similarity among these 14 genes (Figure 1H).

### 3.2 MG-associated prognostic model construction and selection of CCNA2 as a prognostic candidate

**FIGURE 2:**
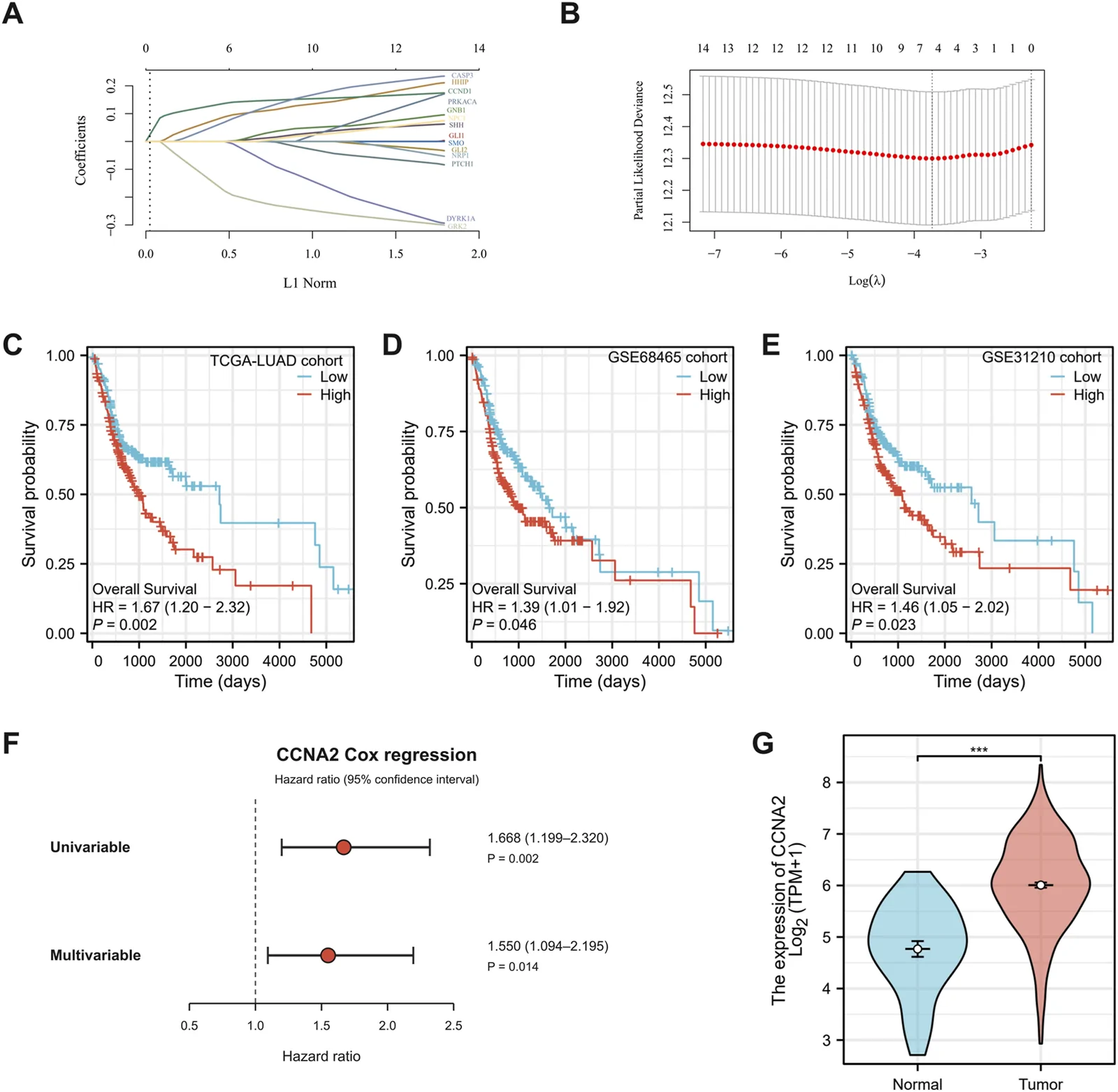
MG-associated prognostic model construction and selection of CCNA2. **(A,B)** LASSO-Cox coefficient paths and ten-fold cross-validation in TCGA-LUAD. **(C–E)** Overall-survival analyses in TCGA-LUAD (n = 329; 164 low and 165 high), GSE68465 (n = 442; 221 low and 221 high), and GSE31210 (n = 226; 113 low and 113 high), respectively. **(F)** Univariable and multivariable Cox regression in TCGA-LUAD (n = 329). **(G)** CCNA2 expression in LUAD tumors (n = 539) and normal lung tissues (n = 59).

In TCGA-LUAD, PTCH1 had the highest mutation frequency among the 14 genes, and enrichment analysis implicated metabolic and immune-related pathways (Supplementary Figures S1A,B). The LASSO-Cox model was constructed using coefficient paths and ten-fold cross-validation (Figures 2A,B) and stratified overall survival in TCGA-LUAD, GSE68465, and GSE31210 (Figures 2C–E); the corresponding ROC analyses are shown in Supplementary Figures S1C–F. The nomogram and calibration analyses are provided in Supplementary Figures S2A,B. Multivariable Cox regression prioritized CCNA2 as a prognostic candidate (Figure 2F), and CCNA2 was upregulated in LUAD tumors (Figure 2G). Co-expression analyses are provided in Supplementary Figures S2C,D.

### 3.3 LUAD tumor microenvironment identification

**FIGURE 3:**
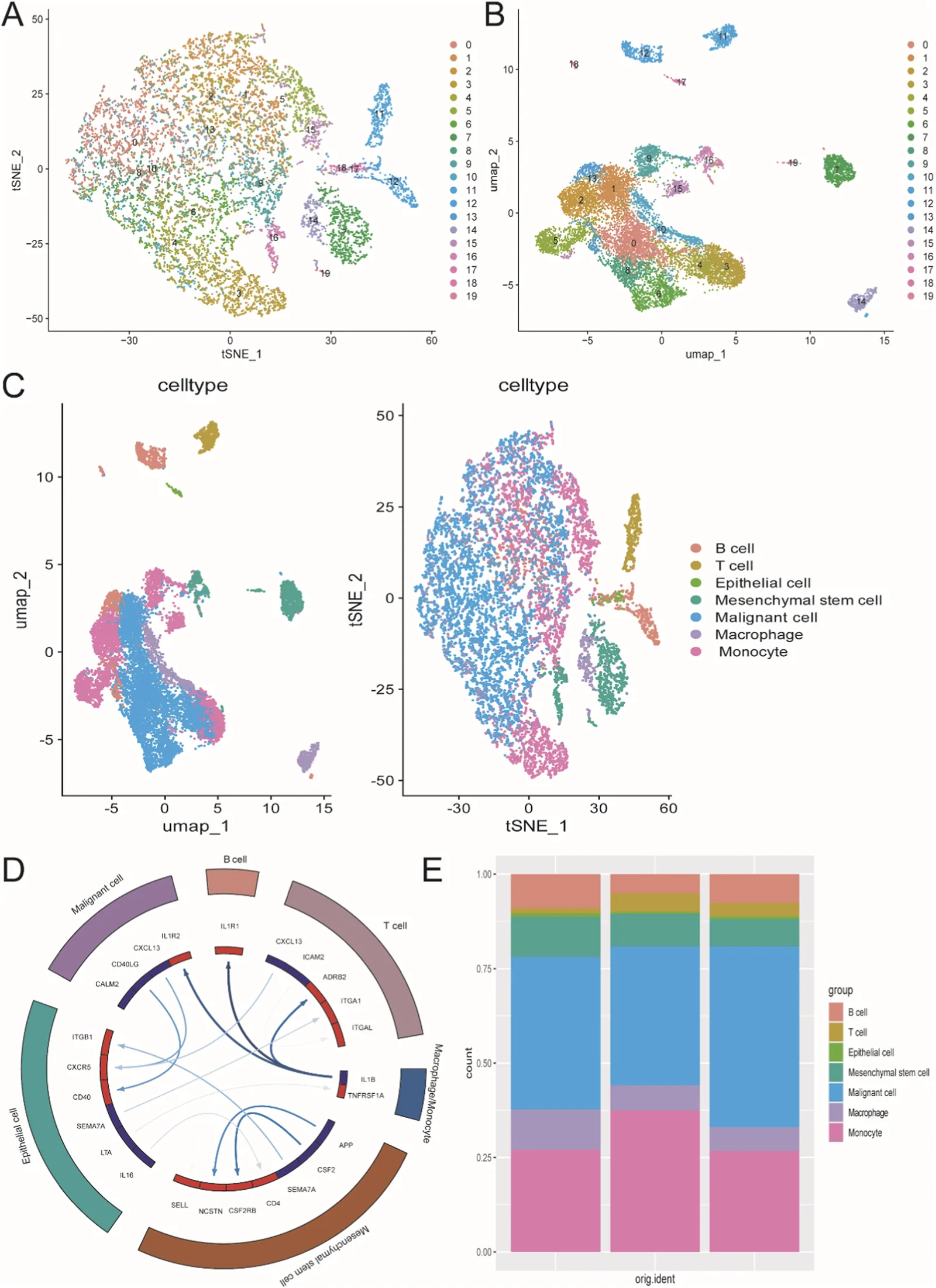
LUAD tumor-microenvironment analysis. Three specimens contributed 11,698 cells after quality control. **(A,B)** t-SNE and UMAP plots of 19 clusters. **(C)** Seven major cell types. **(D)** Inferred ligand-receptor communication. **(E)** Cell-type composition by specimen. Specimens were biological units and cells were nested observations.

Analysis of GSE131907 identified 19 clusters and seven major cell types, and CellChat described inferred intercellular communication patterns among the annotated populations (Figure 3).

### 3.4 Single-cell characterization of the CCNA2 prognostic candidate

**FIGURE 4:**
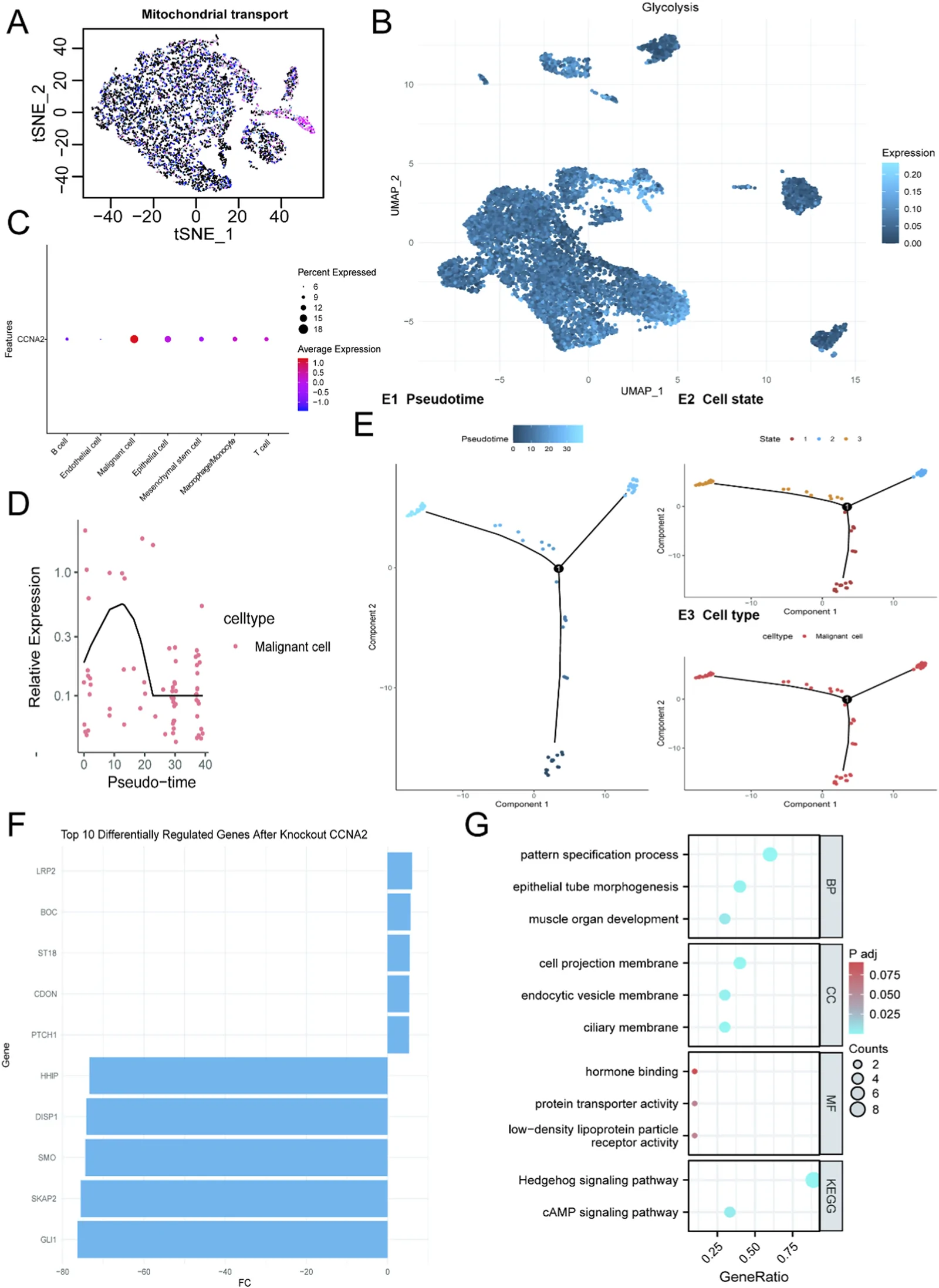
Single-cell characterization of CCNA2. **(A,B)** Mitochondrial-transport and glycolysis activities (10,000 cells). **(C)** CCNA2 expression across annotated populations (11,698 cells). **(D)** CCNA2 expression along pseudotime. **(E1)** Pseudotime; **(E2)** cell state; **(E3)** cell type. **(F,G)** Virtual-knockout and enrichment analyses (4,244 malignant cells; 10 genes for GO and 9 mapped genes for KEGG). Data were derived from three specimens; specimens were biological units and cells were nested observations.

Mitochondrial-transport and glycolysis activities were highest in malignant cells, and CCNA2 was enriched in this population (Figures 4A–C). Monocle2 described a branched trajectory and CCNA2 expression along pseudotime (Figures 4D,E). Virtual CCNA2 knockout predicted 10 altered genes with GO/KEGG enrichment (Figures 4F,G).

### 3.5 Association of the CCNA2 prognostic candidate with LUAD progression

**FIGURE 5:**
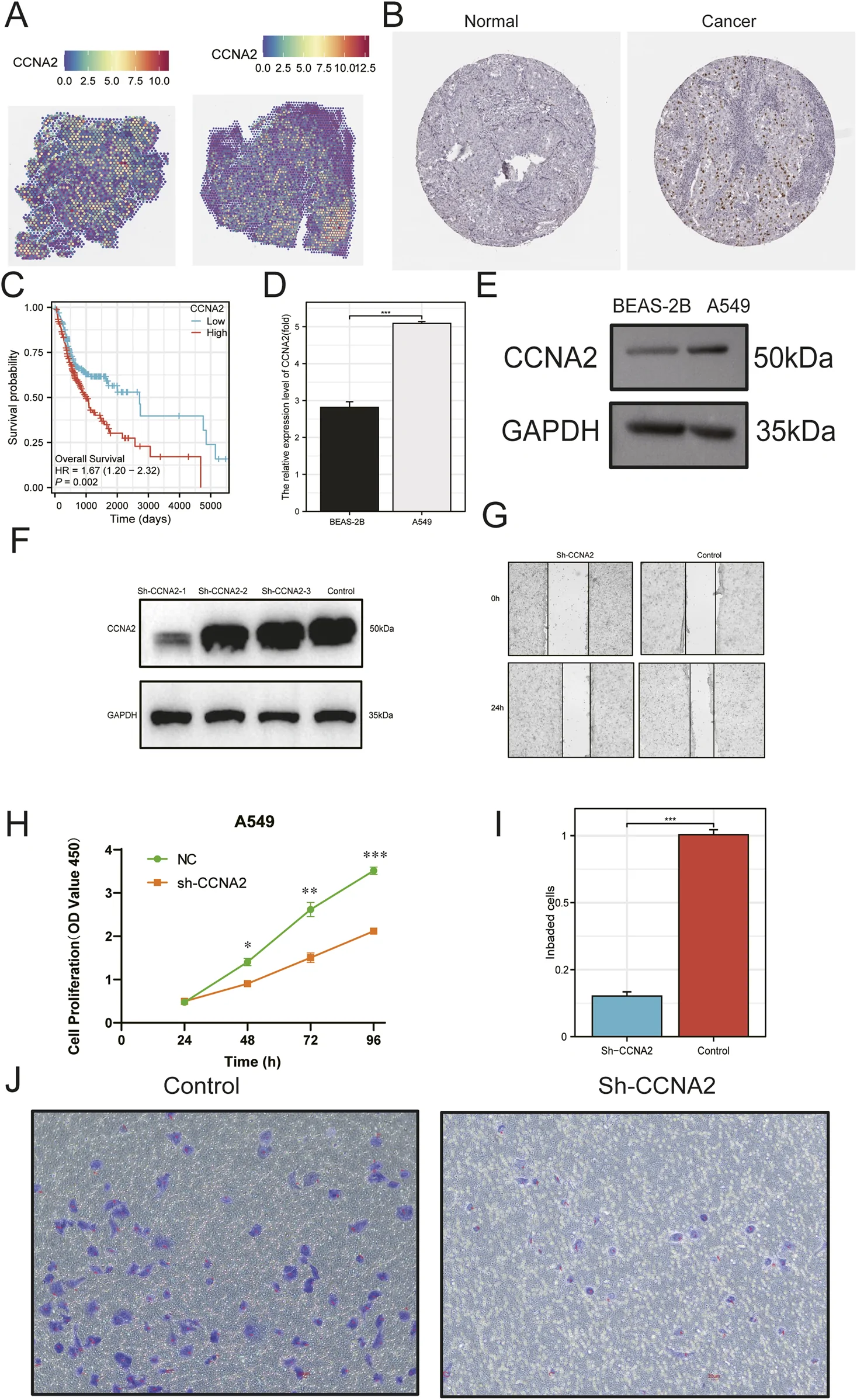
Association of CCNA2 with LUAD progression. **(A)** Spatial CCNA2 expression. **(B)** HPA immunohistochemistry. **(C)** Kaplan-Meier analysis (n = 329; 164 low and 165 high). **(D)** qPCR; each point represents one independent biological experiment (n = 3 per cell line). **(E,F)** Representative blots; labeled images are provided in Supplementary Figures S3, S4. **(G)** Wound healing and **(H)** CCK-8 assays (n = 3 independent biological experiments per group). **(I)** Quantified Transwell invasion; each point represents one independent biological experiment (n = 3 per group). **(J)** Representative Transwell images. Quantitative data are shown as mean ± SD, with individual observations displayed in **(D,I)**.

Spatial transcriptomics and HPA immunohistochemistry supported elevated CCNA2 in LUAD tissues, and high CCNA2 expression was associated with shorter overall survival (Figures 5A–C). The cell-based experiments tested whether CCNA2 was elevated in A549 cells and whether its knockdown reduced malignant phenotypes; all assays used three independent biological experiments per group. Effect estimates, 95% confidence intervals, and P values were as follows: CCNA2 expression was higher in A549 than BEAS-2B cells (difference, 2.27; 95% CI, 1.94–2.60; P = 0.00053; Figures 5D,E). CCNA2 knockdown did not alter CCK-8 absorbance at 24 h (difference, 0.02; 95% CI, −0.03 to 0.07; adjusted P = 0.288), but reductions at 48, 72, and 96 h were 0.50, 1.11, and 1.40, respectively (95% CIs, 0.32–0.68, 0.78–1.45, and 1.21–1.58; all adjusted P ≤ 0.0093; Figure 5H). Wound closure decreased by 11.57 percentage points (95% CI, 6.61–16.53; P = 0.0034; Figure 5G), and normalized invasion decreased by 0.803 (95% CI, 0.751–0.855; P = 2.23 × 10^−6^; Figures 5I,J). Representative blots are shown in Figures 5E,F.

### 3.6 Therapeutic enrichment targeting CCNA2 for LUAD patients

**FIGURE 6:**
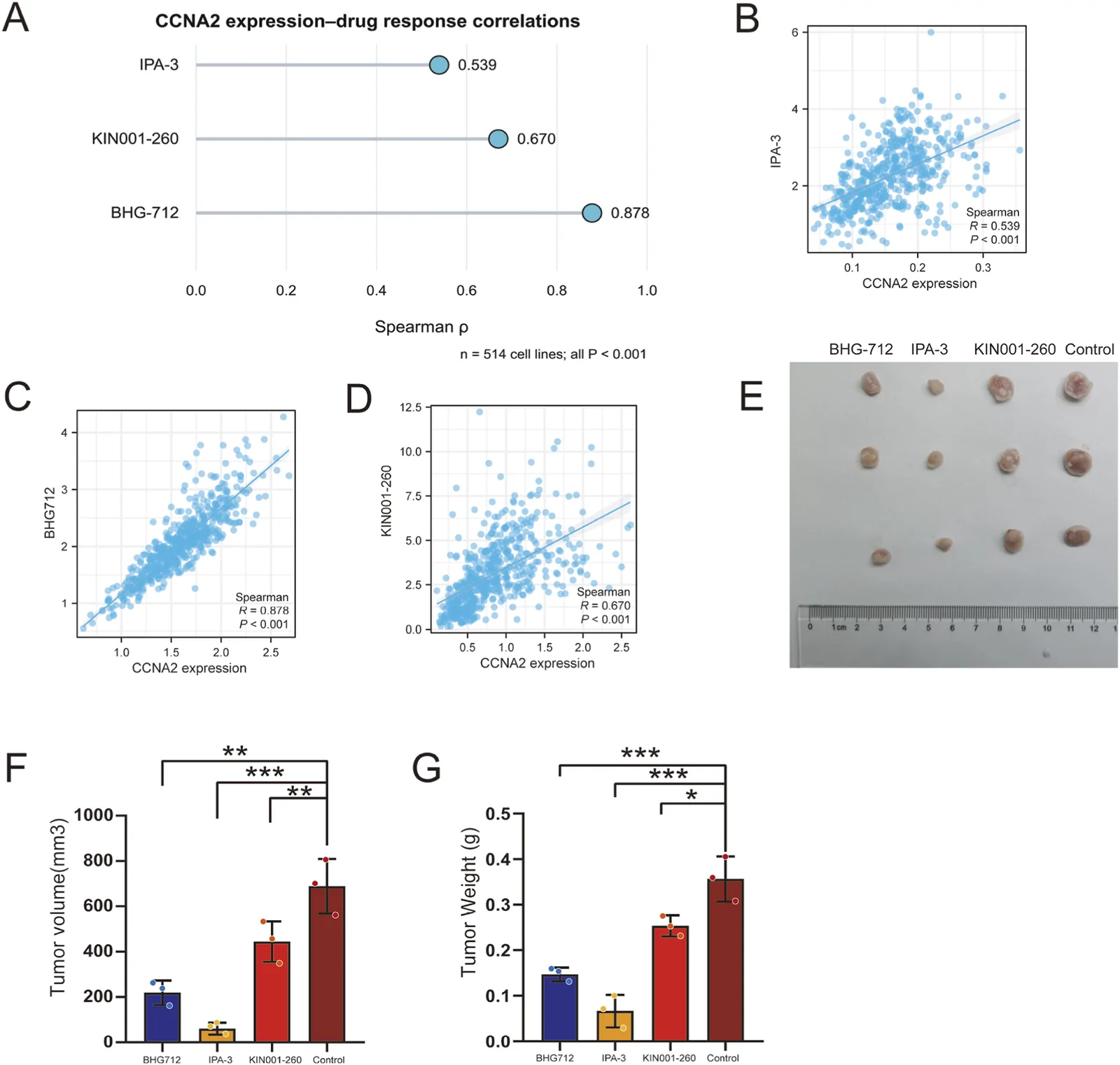
Candidate compounds associated with CCNA2 and their effects in the LUAD xenograft model. **(A)** Correlations are shown as negative after removal of the absolute-value transformation applied to standardize drug-response values. One-dimensional summary of Spearman correlations between CCNA2 expression and drug response for IPA-3, KIN001-260, and BHG-712 (n = 514 GDSC cell lines per drug; all P < 0.001). **(B–D)** Drug-specific scatter plots for IPA-3, BHG-712, and KIN001-260, respectively (n = 514 GDSC cell lines per drug). Each point represents one cell line with paired, non-missing CCNA2-expression and drug-response data. **(E)** Excised tumors after compound or vehicle treatment. Columns indicate the four treatment groups, and each row represents one independent mouse (n = 3 per group); row order has no analytical meaning. **(F,G)** Endpoint tumor volume and weight. Each point represents one independent mouse (n = 3 per group); horizontal lines and error bars show mean ± SD, and brackets show Holm-adjusted comparisons with control.

Using GSCA, we identified BHG-712, IPA-3, and KIN001-260 as candidate compounds associated with CCNA2 expression (Figures 6A–D). The xenograft experiment tested whether these compounds reduced tumor burden relative to vehicle; each group contained three independent mice (Figures 6E–G). IPA-3 produced the largest reductions in tumor volume (625.83 mm^3^; 95% CI, 342.67–908.99; adjusted P = 0.0288) and weight (0.290 g; 95% CI, 0.189–0.391; adjusted P = 0.0053). BHG-712 also reduced volume (469.17 mm^3^; 95% CI, 213.88–724.46; adjusted P = 0.0288) and weight (0.210 g; 95% CI, 0.099–0.321; adjusted P = 0.0242), whereas KIN001-260 did not reach adjusted significance for volume (244.17 mm^3^; 95% CI, −5.03 to 493.37; adjusted P = 0.0529) or weight (0.103 g; 95% CI, −0.0002 to 0.207; adjusted P = 0.0502).

## 4 Discussion and conclusion

In this study, we integrated bulk, single-cell, and spatial transcriptomic analyses with *in vitro* and *in vivo* experiments to characterize a mitochondrial transport- and glycolysis-associated framework in LUAD (Tan et al., 2023). The resulting gene signature stratified patient prognosis, and multivariable Cox analysis prioritized CCNA2 as a prognostic candidate. Single-cell and spatial transcriptomic analyses further characterized the cellular and tissue distribution of CCNA2 (Fang et al., 2022), while CCNA2 knockdown in A549 cells reduced proliferation, wound closure, and invasion. In the xenograft model, IPA-3 produced the greatest reduction in tumor burden among the tested compounds. These findings represent complementary levels of evidence: the computational analyses generated the clinical and molecular hypotheses, the cellular experiments evaluated the functional consequences of CCNA2 knockdown, and the animal experiment assessed the *in vivo* effects of the prioritized compounds. Collectively, these results support further investigation of the MG-associated prognostic framework, CCNA2, and IPA-3 in LUAD.

CCNA2 encodes cyclin A2, a cell-cycle regulator that promotes S-phase entry and G2/M transition (Zhou et al., 2024b). Its dysregulation has been associated with cancer metabolism and progression. Previous studies in breast and prostate carcinoma have linked CCNA2-related pathways to glycolytic phenotypes, providing context for the observed association between CCNA2 and the MG transcriptional signature. In lung cancer, CCNA2 overexpression has been associated with poor prognosis (Yang et al., 2024). Our multi-omic analyses extend these observations by showing CCNA2 expression along malignant-cell trajectories and in spatial transcriptomic specimens; however, these findings remain associative and require direct metabolic and mechanistic validation.

In conclusion, our study establishes a clinically relevant MG-associated molecular model for LUAD, with CCNA2 prioritized as a prognostic candidate linking cell-cycle progression, metabolic dysregulation, and tumor invasion. The prognostic value of the model and the *in vivo* efficacy of IPA-3 offer translational potential for personalized risk assessment and targeted therapy in LUAD. However, several limitations must be acknowledged. First, the MG-associated signature was derived from a curated public-sourced gene list and computational inference from transcriptomic data without performing direct metabolomic profiling or metabolic flux assays, such as Seahorse analysis, lactate quantification, or oxygen consumption rate measurements on LUAD samples or cell lines. Consequently, the MG-associated signature should be interpreted as a transcriptional proxy for mitochondrial transport and glycolysis pathway activity, rather than a direct measure of metabolic flux. Future studies incorporating metabolomics and stable isotope tracing are required to validate the metabolic consequences of CCNA2 dysregulation. Second, while our co-expression analysis provides additional correlative support for the association between CCNA2 and MG-related genes, we did not perform direct biochemical experiments to demonstrate that CCNA2 physically interacts with or regulates specific MT proteins or glycolytic enzymes. Protein-protein interaction studies, enzyme activity assays, and metabolic flux analyses are needed to establish a causal mechanistic link between CCNA2 and the MG-associated pathways. Third, our *in vitro* functional validation was performed exclusively in the A549 LUAD cell line. Given the well-recognized heterogeneity of LUAD, the generalizability of our findings to other LUAD subtypes or additional cell lines, such as H1975, HCC827, H1299, remains to be confirmed. Future studies should validate CCNA2 expression and functional roles in a broader panel of LUAD cell lines. Fourth, IPA-3, the compound prioritized in our drug sensitivity analysis, is primarily recognized as a PAK1 inhibitor (Zhang L. et al., 2025), and its effect on CCNA2-high tumors might be indirect. Although we observed a significant anti-tumor effect of IPA-3 in CCNA2-high tumors in the xenograft model, we did not demonstrate direct binding between IPA-3 and CCNA2, nor did we establish that the therapeutic effect is specifically mediated through CCNA2 rather than PAK1 or other off-target pathways. Future studies employing cellular thermal shift assays, surface plasmon resonance, or CCNA2-specific knockdown-rescue experiments are required to verify the target specificity of IPA-3. Fifth, the single-cell and spatial RNA-seq dataset analysis in this study comprised only 3 and 2 LUAD patient samples respectively. While our findings are consistent with bulk transcriptomic data from larger cohorts, the limited sample size of the single-cell and spatial RNA-seq data may not capture the full heterogeneity of the LUAD microenvironment. Larger single-cell and spatial RNA-seq cohorts are needed to confirm the cellular localization and differentiation dynamics of CCNA2.

## Data Availability

The original contributions presented in the study are publicly available. Bulk and single-cell transcriptomic datasets (GSE17475, GSE12667, GSE140797, GSE68465, GSE31210, and GSE131907) are available from the Gene Expression Omnibus (https://www.ncbi.nlm.nih.gov/geo/). TCGA-LUAD clinical and multi-omics data are available from the Genomic Data Commons (https://portal.gdc.cancer.gov/). Immunohistochemistry images are available from the Human Protein Atlas (https://www.proteinatlas.org/), and drug-response screening information was obtained from GSCA. Numerical source data for Figures 5D,G,H,I, 6F,G, together with sample-size definitions and xenograft study metadata, are provided in Supplementary Data S1.

## References

[B1] Awasthi S. Kumar R. Pradhan D. Rawal N. Goel H. Sahu P. (2024). Genomic landscape of gallbladder cancer: insights from whole exome sequencing. Int. J. Surg. 110 (11), 6883–6897. 10.1097/JS9.0000000000002031 39166960 PMC11573093

[B2] Cao G. Xuan X. Li Y. Hu J. Zhang R. Jin H. (2023). Single-cell RNA sequencing reveals the vascular smooth muscle cell phenotypic landscape in aortic aneurysm. Cell Commun. Signal 21 (1), 113. 10.1186/s12964-023-01120-5 37189183 PMC10184349

[B3] Chang W. Li H. Zhong L. Zhu T. Chang Z. Ou W. (2022). Development of a copper metabolism-related gene signature in lung adenocarcinoma. Front. Immunol. 13, 1040668. 10.3389/fimmu.2022.1040668 36524120 PMC9744782

[B4] Deng Y. Zheng Y. Li D. Hong Q. Zhang M. Li Q. (2021). Expression characteristics of interferon-stimulated genes and possible regulatory mechanisms in lupus patients using transcriptomics analyses. EBioMedicine 70, 103477. 10.1016/j.ebiom.2021.103477 34284174 PMC8318865

[B5] Dias Amoedo N. Dard L. Sarlak S. Mahfouf W. Blanchard W. Rousseau B. (2020). Targeting human lung adenocarcinoma with a suppressor of mitochondrial superoxide production. Antioxid. Redox Signal 33 (13), 883–902. 10.1089/ars.2019.7892 32475148

[B6] Ding L. Getz G. Wheeler D. A. Mardis E. R. McLellan M. D. Cibulskis K. (2008). Somatic mutations affect key pathways in lung adenocarcinoma. Nature 455 (7216), 1069–1075. 10.1038/nature07423 18948947 PMC2694412

[B7] Fang Z. Li J. Cao F. Li F. (2022). Integration of scRNA-Seq and bulk RNA-Seq reveals molecular characterization of the immune microenvironment in Acute pancreatitis. Biomolecules 13 (1), 78. 10.3390/biom13010078 36671463 PMC9855877

[B8] Fernie A. R. Carrari F. Sweetlove L. J. (2004). Respiratory metabolism: glycolysis, the TCA cycle and mitochondrial electron transport. Curr. Opin. Plant Biol. 7 (3), 254–261. 10.1016/j.pbi.2004.03.007 15134745

[B9] Hänzelmann S. Castelo R. Guinney J. (2013). GSVA: gene set variation analysis for microarray and RNA-seq data. BMC Bioinforma. 14, 7. 10.1186/1471-2105-14-7 23323831 PMC3618321

[B10] Hirano Y. Suzuki H. Nakayama J. Yamamoto T. Hirata E. Araya J. (2026). An integrated single-cell lung cancer atlas reveals distinct fibroblast phenotypes between adenocarcinoma and squamous cell carcinomas. NPJ Precis. Oncol. 10 (1), 72. 10.1038/s41698-026-01279-3 41577803 PMC12920623

[B11] Huang Y. Niu Y. Wang X. Li X. He Y. Liu X. (2024). Identification of novel biomarkers related to neutrophilic inflammation in COPD. Front. Immunol. 15, 1410158. 10.3389/fimmu.2024.1410158 38873611 PMC11169582

[B12] Jin H. Wen G. Zhu J. Liu J. Li J. Yao S. (2024). Pantoprazole suppresses carcinogenesis and growth of hepatocellular carcinoma by inhibiting glycolysis and Na(+)/H(+) exchange. Drug Dev. Res. 85 (4), e22198. 10.1002/ddr.22198 38764200

[B13] Kang Z. Y. Huang Q. Y. Zhen N. X. Xuan N. X. Zhou Q. C. Zhao J. (2024). Heterogeneity of immune cells and their communications unveiled by transcriptome profiling in acute inflammatory lung injury. Front. Immunol. 15, 1382449. 10.3389/fimmu.2024.1382449 38745657 PMC11092984

[B14] Langfelder P. Horvath S. (2008). WGCNA: an R package for weighted correlation network analysis. BMC Bioinforma. 9, 559. 10.1186/1471-2105-9-559 19114008 PMC2631488

[B15] Leek J. T. Johnson W. E. Parker H. S. Jaffe A. E. Storey J. D. (2012). The sva package for removing batch effects and other unwanted variation in high-throughput experiments. Bioinformatics 28 (6), 882–883. 10.1093/bioinformatics/bts034 22257669 PMC3307112

[B16] Li F. Liang J. Wei X. (2025). Epigenetic modification of Castor zinc finger 1 (CASZ1) is associated with tumor microenvironments and prognosis of clear cell renal cell carcinoma. Int. J. Surg. 111 (1), 988–997. 10.1097/JS9.0000000000002070 39235847 PMC11745609

[B17] Liang J. Li H. Han J. Jiang J. Wang J. Li Y. (2020). Mex3a interacts with LAMA2 to promote lung adenocarcinoma metastasis *via* PI3K/AKT pathway. Cell Death Dis. 11 (8), 614. 10.1038/s41419-020-02858-3 32792503 PMC7427100

[B18] Liu C. J. Hu F. F. Xie G. Y. Miao Y. R. Li X. W. Zeng Y. (2023). GSCA: an integrated platform for gene set cancer analysis at genomic, pharmacogenomic and immunogenomic levels. Brief. Bioinform 24 (1), bbac558. 10.1093/bib/bbac558 36549921

[B19] Liu W. Xia L. peng Y. Cao Q. Xu K. Luo H. (2025). Unraveling the significance of cuproptosis in hepatocellular carcinoma heterogeneity and tumor microenvironment through integrated single-cell sequencing and machine learning approaches. Discov. Oncol. 16 (1), 900. 10.1007/s12672-025-02696-9 40411678 PMC12103433

[B20] Meng Q. Bao D. Liu S. Huang J. Guo M. Dai B. (2024). ADAM Metallopeptidase domain 19 promotes skin fibrosis in systemic sclerosis *via* neuregulin-1. Mol. Med. 30 (1), 269. 10.1186/s10020-024-01047-8 39716051 PMC11665244

[B21] Neumann J. Feuerhake F. Kayser G. Wiech T. Aumann K. Passlick B. (2010). Gene expression profiles of lung adenocarcinoma linked to histopathological grading and survival but not to EGF-R status: a microarray study. BMC Cancer 10, 77. 10.1186/1471-2407-10-77 20196851 PMC2843676

[B22] Osorio D. Zhong Y. Li G. Xu Q. Yang Y. Tian Y. (2022). scTenifoldKnk: an efficient virtual knockout tool for gene function predictions *via* single-cell gene regulatory network perturbation. Patterns (N Y) 3 (3), 100434. 10.1016/j.patter.2022.100434 35510185 PMC9058914

[B23] Passarella S. Schurr A. Portincasa P. (2021). Mitochondrial transport in glycolysis and gluconeogenesis: achievements and perspectives. Int. J. Mol. Sci. 22 (23), 12620. 10.3390/ijms222312620 34884425 PMC8657705

[B24] Ritchie M. E. Phipson B. Wu D. Hu Y. Law C. W. Shi W. (2015). Limma powers differential expression analyses for RNA-sequencing and microarray studies. Nucleic Acids Res. 43 (7), e47. 10.1093/nar/gkv007 25605792 PMC4402510

[B25] Shen A. Zheng S. Tang X. Yao Y. Yin E. Sun N. (2026). Integrated multi-omics analyses identified the H3K18la-based spatiotemporal characteristics and risk-stratified treatment strategy in lung adenocarcinoma. Cancer Lett. 638, 218158. 10.1016/j.canlet.2025.218158 41274397

[B26] Shu J. Jiang J. Zhao G. (2023). Identification of novel gene signature for lung adenocarcinoma by machine learning to predict immunotherapy and prognosis. Front. Immunol. 14, 1177847. 10.3389/fimmu.2023.1177847 37583701 PMC10424935

[B27] Su M. C. Lee A. M. Zhang W. Maeser D. Gruener R. F. Deng Y. (2024). Computational modeling to identify drugs targeting metastatic castration-resistant prostate cancer characterized by heightened glycolysis. Pharm. (Basel) 17 (5), 569. 10.3390/ph17050569 38794139 PMC11124089

[B28] Sun Q. Zheng S. Tang W. Wang X. Wang Q. Zhang R. (2024). Prediction of lung adenocarcinoma prognosis and diagnosis with a novel model anchored in circadian clock-related genes. Sci. Rep. 14 (1), 18202. 10.1038/s41598-024-68256-3 39107445 PMC11303802

[B29] Tan Z. Chen X. Zuo J. Fu S. Wang H. Wang J. (2023). Comprehensive analysis of scRNA-Seq and bulk RNA-Seq reveals dynamic changes in the tumor immune microenvironment of bladder cancer and establishes a prognostic model. J. Transl. Med. 21 (1), 223. 10.1186/s12967-023-04056-z 36973787 PMC10044739

[B30] Wang S. Wang R. Hu D. Zhang C. Cao P. Huang J. (2024). Machine learning reveals diverse cell death patterns in lung adenocarcinoma prognosis and therapy. NPJ Precis. Oncol. 8 (1), 49. 10.1038/s41698-024-00538-5 38409471 PMC10897292

[B31] Wang S. Wang Y. Gan M. Wan L. Liu Y. Xu Y. (2025). Bioinformatics analysis of oxidative phosphorylation-related differentially expressed genes in osteoporosis. Eur. J. Med. Res. 30 (1), 294. 10.1186/s40001-025-02568-6 40241169 PMC12001448

[B32] Wei X. Li X. Hu S. Cheng J. Cai R. (2023). Regulation of ferroptosis in lung adenocarcinoma. Int. J. Mol. Sci. 24 (19), 14614. 10.3390/ijms241914614 37834062 PMC10572737

[B33] Wei X. Wang T. Xing Z. Shi Q. Gu J. Fan Q. (2025). Sirtuin 3 protects lung adenocarcinoma from ferroptosis by deacetylating and stabilizing mitochondrial glutamate transporter solute carrier family 25 member A22. Antioxidants (Basel) 14 (4), 403. 10.3390/antiox14040403 40298644 PMC12024224

[B34] Yang S. Du J. Wang W. Zhou D. Xi X. (2024). APOC1 is a prognostic biomarker associated with M2 macrophages in ovarian cancer. BMC Cancer 24 (1), 364. 10.1186/s12885-024-12105-z 38515073 PMC10956310

[B35] Yu G. Li F. Qin Y. Bo X. Wu Y. Wang S. (2010). GOSemSim: an R package for measuring semantic similarity among GO terms and gene products. Bioinformatics 26 (7), 976–978. 10.1093/bioinformatics/btq064 20179076

[B36] Yu G. Wang L. G. Han Y. He Q. Y. (2012). clusterProfiler: an R package for comparing biological themes among gene clusters. Omics 16 (5), 284–287. 10.1089/omi.2011.0118 22455463 PMC3339379

[B37] Zhang W. Qu H. Ma X. Li L. Wei Y. Wang Y. (2023). Identification of cuproptosis and immune-related gene prognostic signature in lung adenocarcinoma. Front. Immunol. 14, 1179742. 10.3389/fimmu.2023.1179742 37622116 PMC10445162

[B38] Zhang D. Li Y. Liu T. Liu X. Zhang J. (2025a). Neutrophil-Related Genes predict prognosis and contribute to immunosuppression in acute Myeloid leukemia. Blood Lymphat. Cancer 15, 115–132. 10.2147/BLCTT.S529074 40859962 PMC12372834

[B39] Zhang L. Wang S. Wang L. (2025b). Comprehensive analysis pinpoints CCNA2 as a prognostic and immunological biomarker in non-small cell lung cancer. BMC Pulm. Med. 25 (1), 14. 10.1186/s12890-025-03490-7 39799294 PMC11725219

[B40] Zhou Y. Peng X. Fang C. Peng X. Tang J. Wang Z. (2024a). Histones methyltransferase NSD3 inhibits lung adenocarcinoma glycolysis through interacting with PPP1CB to decrease STAT3 signaling pathway. Adv. Sci. (Weinh) 11 (38), e2400381. 10.1002/advs.202400381 39119928 PMC11481231

[B41] Zhou H. Y. Wang Y. C. Wang T. Wu W. Cao Y. Y. Zhang B. C. (2024b). CCNA2 and NEK2 regulate glioblastoma progression by targeting the cell cycle. Oncol. Lett. 27 (5), 206. 10.3892/ol.2024.14339 38516683 PMC10956385

[B42] Zhou J. Jiang Y. Yu M. Wang M. Li Y. Ji D. (2026). Kinic index: an artificial intelligence-driven predictive model and multitarget drug discovery framework for hepatocellular carcinoma patients. NPJ Precis. Oncol. 10 (1), 132. 10.1038/s41698-026-01324-1 41691080 PMC13018533

