# Supplementary material for: Identification and validation of mitochondrial transport and glycolysis-associated prognostic and therapeutic target for lung adenocarcinoma patients
Source: Front Genet. 2026 Aug 27;17:1924235. doi: 10.3389/fgene.2026.1924235 (PMC13557699; doi:10.3389/fgene.2026.1924235)
Supplement: Supplementary file 1 [file Supplementaryfile1.docx]

**Supplementary Figures**

**Supplementary Figure S1**

Supplementary Figure S1. Extended prognostic analyses. (A) Oncoplot of the 14 input genes (n=38 altered cases). (B) GO and KEGG enrichment. (C) Training ROC analysis. (D-F) Time-dependent ROC analyses in TCGA-LUAD (n=329), GSE68465 (n=442), and GSE31210 (n=226), respectively.

**Supplementary Figure S2**

Supplementary Figure S2. Nomogram, calibration, and co-expression analyses. (A-B) Nomogram and calibration analyses in TCGA-LUAD (n=329). (C-D) Co-expression analyses for mitochondrial transport- and glycolysis-associated genes (n=330 tumors).

Supplementary Figure S3

**Supplementary Figure S3.** Labeled immunoblot images corresponding to Figure 5E. CCNA2 (40 kDa) and GAPDH (35 kDa) were detected in BEAS-2B and A549 cells. Lane identities are shown above each image. The image is representative of three independent biological experiments and was not used for inferential testing.

Supplementary Figure S4

**Supplementary Figure S4.** Labeled immunoblot images corresponding to Figure 5F. For the CCNA2 image, lane order is M (molecular-weight marker), sh-CCNA2-1, sh-CCNA2-2, sh-CCNA2-3, and sh-Control. The GAPDH image contains sh-CCNA2-1, sh-CCNA2-2, sh-CCNA2-3, and sh-Control in the labeled order. The image is representative of three independent biological experiments and was not used for inferential testing.
